# Biomarker Integration in the Perioperative Management of Early-Stage Non-Small-Cell Lung Cancer

**DOI:** 10.3390/jcm15197644

**Published:** 2026-10-02

**Authors:** Emily P. Rabinovich, Linda W. Martin

**Affiliations:** 1 Department of Surgery, University of Virginia Health System, Charlottesville, VA 22903, USA; epr3wxp@uvahealth.org; 2 Division of Cardiothoracic Surgery, Department of Surgery, University of Virginia Health System, Charlottesville, VA 22903, USA

**Keywords:** non-small-cell lung cancer, driver mutations, precision oncology, biomarker-driven therapy, perioperative therapy

## Abstract

The concept of personalized medicine has tremendous appeal—tailoring diagnosis and treatment to an individual, rather than a “one-size-fits-all” approach. The management of non-small-cell lung cancer (NSCLC) has rapidly evolved toward strategies increasingly guided by tumor biology and precision oncology. The safety and significant efficacy of biomarker-directed therapies in metastatic disease have fueled interest in applying precision oncology to early-stage, operable NSCLC. Advances in molecular profiling have revealed that a substantial proportion of resected NSCLC harbor actionable driver mutations, enabling the integration of targeted therapies into the perioperative setting. The success of adjuvant epidermal growth factor receptor and anaplastic lymphoma kinase inhibition established a framework for biomarker-driven therapy and highlights the importance of comprehensive molecular testing at diagnosis. In parallel, perioperative immune checkpoint inhibitor-based strategies have expanded treatment options for appropriately selected patients, further emphasizing the importance of molecular profiling. Significant implementation gaps remain, including incomplete molecular testing, delayed results, inadequate tissue sampling, fragmented workflows, and disparities in access. Beyond clinically actionable driver mutations, emerging research highlights radiogenomics and advances in the use of circulating tumor DNA for minimal residual disease detection, with important implications for early recurrence detection and patient prognostication, although these technologies differ in clinical maturity. Collectively, these developments emphasize the molecular heterogeneity underlying early-stage NSCLC and the expanding role of biomarkers in guiding clinical decision-making. Integrating genomic, radiographic, and biologic markers into clinical workflows will be essential to refine risk stratification, personalize perioperative treatment strategies, and ultimately improve long-term oncologic outcomes.

## 1. Introduction

The management of early-stage non-small-cell lung cancer (NSCLC) is undergoing a fundamental transformation as advances in molecular profiling continue to reshape therapeutic decision-making. Surgical resection remains the cornerstone of treatment for stage I–III disease, with postoperative management traditionally guided by pathologic stage and selected clinicopathologic risk factors. While this approach has improved survival, recurrence remains a major challenge, particularly among patients with more advanced pathologic stage. Among patients who develop recurrence following complete (R0) resection, distant recurrence is common and continues to account for substantial disease-related mortality [1,2,3]. Thus, the management of resectable NSCLC has since evolved beyond purely anatomic staging to incorporate molecular biomarkers and immune checkpoint inhibitor (ICI) therapy [4].

Recent advances in molecular oncology have contributed to the transformation of the treatment paradigm for early-stage NSCLC. The identification of oncogenic driver mutations in genes such as epidermal growth factor receptor (EGFR), anaplastic lymphoma kinase (ALK), and Kirsten Rat Sarcoma (KRAS) has allowed for the development of targeted therapies that improve response rates and progression-free survival and reduce toxicity in patients with advanced NSCLC relative to traditional chemotherapy [5,6]. Landmark trials, such as the ADAURA trial, have established that oncogenic drivers, such as EGFR mutations, carry predictive significance in the adjuvant setting, with patients demonstrating superior disease-free survival (DFS) and overall survival (OS) [7,8]. In resected disease, the number of alterations with established perioperative actionability remains far smaller than the number identified by comprehensive sequencing techniques. To date, select drivers have level 1 evidence in this setting: ADAURA established disease-free and overall survival benefits with adjuvant osimertinib for sensitizing EGFR-mutated NSCLC, ALINA established major disease-free survival benefit with adjuvant alectinib for ALK-rearranged disease, and LIBRETTO-432 extended this paradigm to adjuvant selpercatinib for RET-fusion-positive disease [4,9]. The prevalence of oncogenic driver alterations in NSCLC varies considerably according to histology, smoking status, testing platform, and geographic variation. Molecular profiling has revealed that up to 70% of resected NSCLC cases harbor actionable driver mutations [2,10,11,12]. In this setting, the term “actionable” requires clinical context. Broad next-generation sequencing may identify alterations for which targeted therapies are available in advanced disease, but this should not be interpreted as evidence that all have validated perioperative targeted therapies. At present, sensitizing EGFR mutations and ALK rearrangements have the strongest phase III evidence supporting genotype-directed adjuvant therapy after complete resection.

In Western and European lung adenocarcinoma cohorts, EGFR-sensitizing oncogenic driver mutations have been detected in approximately 10–15% of fully genotyped tumors. Population-level estimates are substantially higher in Asian than European cohorts, particularly among patients with adenocarcinoma and never smokers; therefore, rates derived from these highly selected cohorts should not be generalized to all resectable NSCLC [13,14]. As a result, NSCLC is increasingly recognized as a molecularly heterogeneous disease, in which these molecular features and driver mutations may significantly influence patient prognosis, recurrence risk, and therapeutic decision-making. This evolving understanding of the disease underscores a crucial shift in the management of early-stage NSCLC. As advances in molecular profiling become integrated into routine clinical practice, NSCLC management is increasingly guided by precision oncology, enabling individualized risk stratification, perioperative therapeutic interventions, and improved long-term oncologic outcomes.

This work critically examines the integration of biomarker evaluation in resectable NSCLC through three clinically distinct questions: (1) which biomarkers currently alter perioperative management; (2) how molecular testing should be operationalized across the diagnostic and surgical pathway; and (3) which emerging biomarkers remain investigational despite strong biologic or prognostic associations. Specifically, the authors distinguish established therapeutic biomarkers from future targets, examine implementation disparities, and critically assess the readiness of radiogenomics and ctDNA-based minimal residual disease (MRD) for clinical decision-making. From a surgical perspective, molecular characterization increasingly influences the timing and selection of systemic therapy, but does not yet alter the scope of pulmonary resection or nodal assessment. Priority was given to pivotal randomized trials, prospective studies, major meta-analyses, consensus or guideline documents, and primary studies directly relevant to resectable NSCLC. Studies of advanced disease were included selectively where necessary to provide historical or biologic context.

## 2. EGFR and the Emergence of Biomarker-Driven Precision Therapies for NSCLC

The EGFR experience provides a formative example of the importance of biomarker selection in NSCLC. First-generation EGFR tyrosine kinase inhibitors initially entered clinical use in molecularly unselected advanced NSCLC [15,16,17]. The subsequent identification of activating EGFR mutations in 2004 explained the marked heterogeneity in therapeutic response and transformed EGFR-TKI therapy from an empiric strategy into a biomarker-driven approach [18,19,20,21,22]. This historical sequence established the conceptual framework for the subsequent extension of molecularly directed therapy into resectable disease.

More recently, the ADAURA trial, a phase III, double-blind randomized study, established osimertinib as standard of care for resected EGFR-mutated NSCLC, demonstrating sustained 4-year disease-free survival benefits (stage II–IIIA 70% versus 29%; HR 0.23 (95% CI 0.18–0.30); overall population 73% versus 38%; HR 0.27 (95% CI 0.21–0.34)) [23]. Importantly, the planned final overall-survival analysis subsequently demonstrated that the DFS benefit translated into improved survival (Table 1). Five-year overall survival was 85% with osimertinib versus 73% with placebo among patients with stage II–IIIA disease (HR for death 0.49; 95.03% CI, 0.33–0.73) and 88% versus 78%, respectively, in the overall stage IB–IIIA population (HR 0.49; 95.03% CI, 0.34–0.70) [24]. These findings established that the clinical impact of adjuvant molecular selection extends beyond delaying recurrence.

Osimertinib is a third-generation EGFR TKI with clinically significant activity in the central nervous system (CNS). This property becomes particularly relevant given the propensity for CNS recurrence in EGFR-mutant NSCLC. Rates of brain metastases have been reported in approximately 25 to 40% of patients with EGFR-mutant NSCLC at the time of initial diagnosis for advanced disease populations [25]. This CNS benefit became evident in the ADAURA trial, in which adjuvant Osimertinib markedly reduced CNS recurrence after complete resection of EGFR-mutant stage II to IIIA NSCLC (HR for CNS disease recurrence or death 0.24 (95%CI 0.14–0.42, 2022 updated analyses) [7]. This clinical effect is biologically supported by osimertinib’s improved blood–brain barrier penetration compared with earlier-generation EGFR-TKIs. In preclinical comparative studies, Osimertinib demonstrated the lowest efflux transporter liability and highest free brain-to-free plasma ratio among tested EGFR TKIs, supporting its enhanced CNS exposure [26].

The magnitude of ADAURA’s benefit should be considered alongside unresolved questions regarding optimal treatment duration, toxicity, adherence, cost, and identifying those patients for whom these therapies would confer the greatest benefit. Importantly, an exploratory 8-year survival landmark analysis of ADAURA demonstrated persistence of OS benefit well beyond completion of the planned 3-year treatment period. Among patients with II–IIIA disease, the 8-year OS rate was 74% with osimertinib versus 58% with placebo (HR 0.53, 95% CI 0.38–0.75); in the overall stage IB–IIIA population, 8-year OS was 79% versus 64%, respectively (HR 0.52, 95% CI 0.39–0.71) [27]. Although the OS benefit confirms meaningful clinical efficacy, de-escalation strategies for individual patients should consider the extent to which prolonged kinase inhibition demonstrates durability of survival benefit after treatment completion and suppresses recurrence.

The phase III NeoADAURA trial further moves EGFR-directed therapy into the preoperative setting. Among 358 patients with resectable EGFR-mutated stage II–IIIB NSCLC, major pathologic response occurred in 26% with neoadjuvant osimertinib plus chemotherapy and 25% with osimertinib monotherapy compared with 2% with chemotherapy alone [28]. These findings move molecular testing from a postoperative consideration to a prerequisite for informed preoperative multidisciplinary planning and place greater importance on obtaining adequate tissue from the initial diagnostic biopsy.

The discovery of EGFR mutations fundamentally reshaped the management of resectable lung cancer, shifting the landscape of adjuvant therapy from one historically managed by uniform cytotoxic chemotherapy to one that requires comprehensive molecular profiling to guide targeted precision therapy. The success following EGFR discovery paved the way for further investigations into driver mutations, leading to the discovery of ALK, among others, with their corresponding targeted therapies, which are now routinely incorporated into modern treatment strategies [6,29].

## 3. Beyond EGFR: Expanding the Targetable Landscape

While EGFR provided the framework and remains the most common targetable mutation with an extensive therapeutic armamentarium, the molecular landscape of resectable NSCLC continues to broaden. Anaplastic lymphoma kinase (ALK) rearrangements were discovered in 2007 and are present in approximately 3 to 7% of NSCLC cases [5,30]. The favorable survival associated with modern ALK-directed therapy has largely been demonstrated in advanced ALK-positive disease. In the phase III ALINA trial, patients with completely resected ALK-positive stage IB tumors greater than or equal to 4 cm in size to stage IIIA NSCLC were randomized to adjuvant alectinib for 24 months versus platinum-based chemotherapy. Alectinib significantly improved DFS (HR for disease recurrence or death of 0.24; 95% CI, 0.13 to 0.45; *p* < 0.001) [31]. Importantly, patients treated with adjuvant alectinib demonstrated a marked reduction in CNS recurrence (HR for CNS DFS 0.22; 95% CI 0.08–0.58), paralleling the ADAURA experience with osimertinib in EGFR-mutant disease [31,32]. Overall-survival data remained immature in the primary ALINA report, emphasizing the need for continued follow-up despite the substantial DFS and CNS-DFS effects. EGFR and ALK should therefore be distinguished from a broader group of molecular alterations that are therapeutically actionable in advanced disease but do not yet have equivalent established perioperative utility.

**Table 1:** Selected Phase III Biomarker-Directed Trials in Resectable NSCLC.

Trial	Population	Intervention	Key Findings	CNS Outcomes	Reference
ADAURA	Completely resected EGFR exon 19 deletion/L858R stage IB–IIIA NSCLC	Adjuvant osimertinib up to 3 years versus placebo	4-year DFS: 73% versus 38% overall; final 5-year OS: 88% versus 78% overall; OS HR 0.49	CNS DFS HR 0.24 (95%CI 0.14–0.42) for stage II–IIIA (2022 Updated); 0.18 (95% CI 0.10–0.33) for overall stage IB-IIIA population (2020 Primary)/0.36 (2022 Updated); Exploratory 8-year OS landmark update: 79% versus 64% overall (stage IB–IIIA), OS HR 0.52 (95% CI 0.39–0.71); 74% versus 58% (stage II–IIIA), OS HR 0.53 (95% CI 0.38–0.75) (2026).	[7,23,24,27]
ALINA	Completely resected ALK-positive stage IB ≥ 4 cm-IIIA NSCLC	Adjuvant alectinib for 2 years versus platinum chemotherapy	Stage II–IIIA 2-year DFS: 93.8% versus 63.0%; HR 0.24	CNS DFS HR 0.22	[31]
NeoADAURA	Resectable EGFR-mutated stage II–IIIB NSCLC	Neoadjuvant osimertinib + chemotherapy, osimertinib alone, or chemotherapy	MPR: 26%, 25%, and 2%, respectively	Not the principal reported endpoint	[28]

In addition to EGFR and ALK mutations, KRAS mutations are among the most common oncogenic drivers in NSCLC, occurring in approximately 25–35% of cases in Western populations [33,34]. Despite this prevalence, the clinical benefit of currently available targeted therapies has been more modest in this subgroup of patients compared with other driver mutations, with KRAS G12C inhibitors demonstrating progression-free survival of roughly five to six months among patients with advanced disease [35]. Additional actionable driver mutations, including BRAF V600E, MET exon 14, RET fusions, and NTRK rearrangements, and HER2 (ERBB2) TK domain mutations, are increasingly incorporated into comprehensive next-generation sequencing panels [12,36].

HER2, in particular, illustrates an important distinction between a validated, actionable drug target in metastatic NSCLC and an established, evidence-based target in the perioperative “curative-intent” setting for resectable disease. Activating HER2 TK domain mutations occur in approximately 2–4% of NSCLC, arising predominantly in adenocarcinomas, never-smokers, and women [37]. Zongertinib demonstrated substantial activity in previously treated advanced HER2 TK domain-mutated NSCLC, with an objective response rate of 71% and median progression-free survival of 12.4 months, and received FDA accelerated approval for that setting in 2025 [38]. However, perioperative efficacy has not yet been established, and no HER2-directed agent currently carries a perioperative indication in NSCLC management. Its ongoing adjuvant evaluation illustrates how targets validated in advanced NSCLC are progressively moving into curative-intent trials.

Comprehensive sequencing may additionally detect co-occurring alterations such as TP53, STK11, and KEAP1, which may provide prognostic information and potentially refine the understanding of tumor biology and immune responses [39]. However, their role in selecting perioperative therapy remains considerably less established than the predictive utility of EGFR mutations or ALK rearrangements, as none are yet formally validated, guideline-endorsed predictive biomarkers at their current stages.

## 4. Immune Biomarkers and the Contemporary Perioperative Immunotherapy Landscape

Biomarker-driven perioperative care is not synonymous with targeted therapy, as has been discussed. PD-L1 by immunohistochemistry remains the most routinely measured immune biomarker in NSCLC. However, its predictive performance in the perioperative setting is imperfect, and benefit from chemoimmunotherapy is not uniformly restricted to PD-L1-positive tumors [40,41]. The modern treatment landscape therefore requires assessment of molecular driver status and immune biomarkers to be interpreted together.

CheckMate 816 established the efficacy of neoadjuvant nivolumab plus platinum-based chemotherapy, demonstrating improvements in event-free survival and pathologic complete response compared with chemotherapy alone [42,43]. Subsequent phase III trials expanded this paradigm to perioperative therapy: KEYNOTE-671 demonstrated benefit with neoadjuvant chemotherapy plus pembrolizumab followed by adjuvant pembrolizumab; AEGEAN demonstrated improved event-free survival and pathologic complete response with perioperative durvalumab; and CheckMate 77T demonstrated improved event-free survival and pathologic response with perioperative nivolumab [44,45,46]. Importantly, these trials, among others, all showed that the magnitude of benefit scales with PD-L1, but is not confined to positive tumors. The STS expert consensus states that while PD-L1 predicts response, lack thereof should not exclude patients from consideration of neoadjuvant immunotherapy [40].

These trials also highlight an important practical role for early genotyping. Patients with established sensitizing EGFR mutations or ALK rearrangements represent biologically distinct populations for whom genotype-directed treatment pathways are available and for whom the benefit of perioperative immunotherapy is less clearly established, as certain pivotal immunotherapy trials either explicitly excluded known EGFR and ALK mutations as prespecified eligibility criteria or had a small percentage of representation. Consequently, clinicians should be aware of clinically relevant driver status before initiating perioperative immunotherapy whenever feasible and when the result would alter treatment selection. Pathologic complete response and major pathologic response have also become increasingly important trial endpoints in the neoadjuvant era. Although these measures provide early evidence of antitumor activity, they should not be viewed as complete substitutes for event-free and overall survival when evaluating the durability of perioperative treatment strategies [47].

## 5. Radiogenomics: Investigational Promise, Current Performance, and Limitations

**Figure 1:**
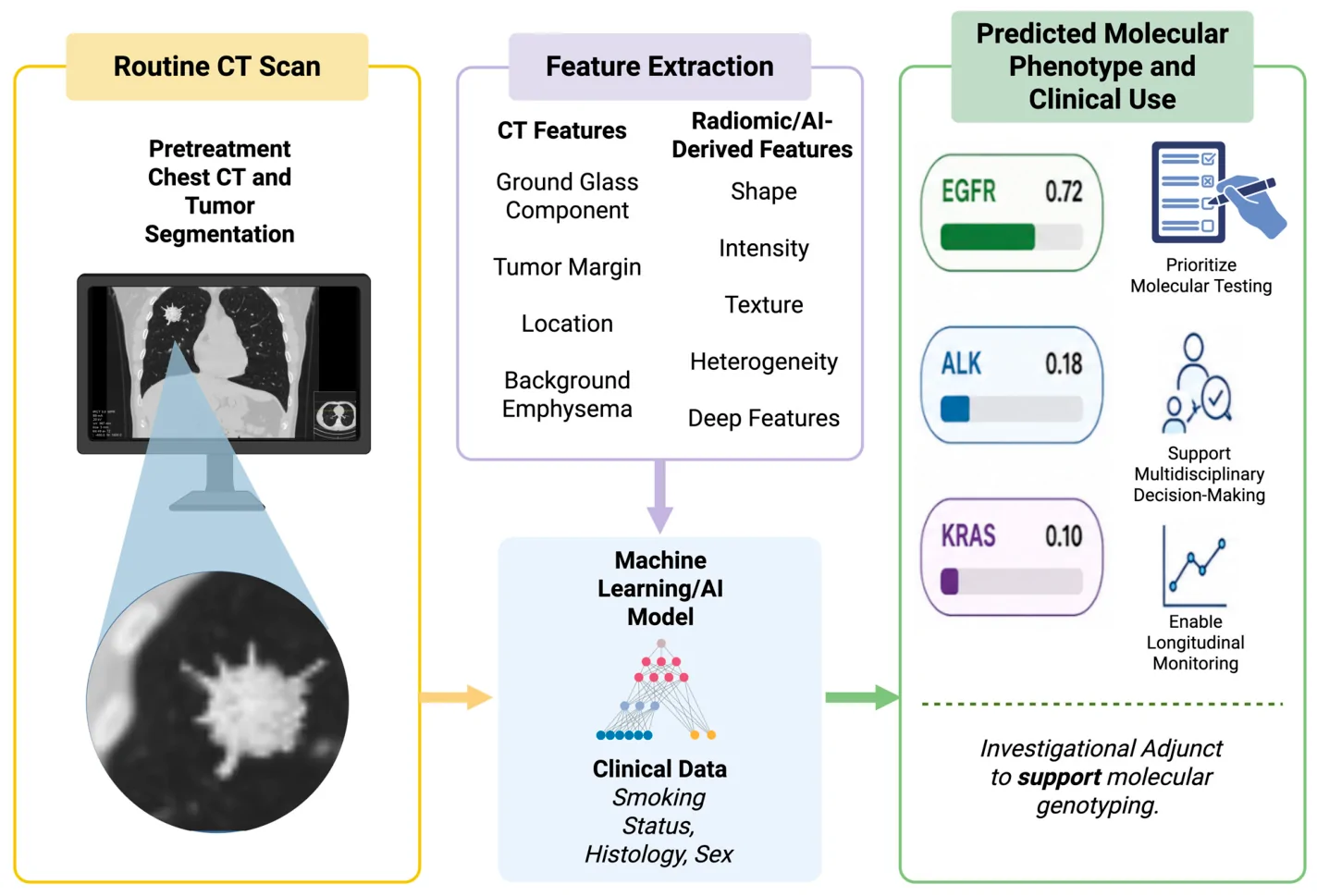
Computed tomography (CT)-based radiogenomics in non-small-cell lung cancer. Routine chest CT can be utilized to derive discrete imaging features and quantitative radiomic data from segmented primary tumors, which, combined with clinical variables, may be analyzed to generate preliminary, noninvasive predictions of oncogenic driver status, such as EGFR or KRAS. Radiogenomic approaches remain investigational, and no validated radiogenomic tools currently replace tissue- or plasma-based profiling. Created in BioRender. Rabinovich, E. (2026) https://BioRender.com/6lepsvc (Accessed on 16 August 2026).

An emerging area of research within this space is radiogenomics, which integrates quantitative imaging features from standard imaging such as computed tomography (CT), positron-emission tomography (PET), or magnetic resonance imaging, with tumor genomic characteristics [48]. The most established and widely studied form of radiogenomics implemented in clinical applications is CT-based radiogenomics, which uses machine learning and artificial intelligence to analyze quantitative features such as texture, shape, image heterogeneity, and attenuation patterns to correlate them with underlying genomic alterations [49,50,51] (Figure 1). This technology has shown promise for non-invasive identification and characterization of EGFR and KRAS mutation status, which may help guide treatment algorithms [51,52].

Published models have demonstrated potentially useful discrimination for common oncogenic alterations, particularly EGFR mutations; however, reported performance varies substantially across datasets and study designs. Representative studies have reported receiver-operating-characteristic area-under-the-curve values broadly ranging from approximately 0.7 to 0.9 for EGFR prediction. Still, this discrimination should not be equated with prospective clinical validity [53,54]. Several methodological limitations currently restrict translation into clinical practice. Much of the radiogenomic literature remains retrospective, frequently single-center, and dependent on relatively small datasets. Scanner manufacturer, acquisition protocol, reconstruction parameters, image segmentation, feature extraction, and preprocessing can substantially influence radiomic features. Model overfitting, incomplete prospective external validation, poor calibration, and performance degradation across institutions remain important concerns [55]. Methodologically, integrating radiographic, molecular, and clinical features represents a multimodal data science problem. Broader multimodal-learning literature highlights additional barriers including modality heterogeneity, missing data, limited interpretability, fairness across demographic groups, and cross-institutional distribution shift—all of which are relevant to radiogenomic implementation [56,57].

A related, investigational form of radiogenomics uses ligand-based, genotype-directed molecular PET radiotracers designed to noninvasively interrogate oncogenic driver status. EGFR-directed radiotracers such as [11C]Erlotinib, [11C]PD153035, and [18F]F-MPG, as well as KRAS-targeted radiotracers such as [18F]PFPMD, have been evaluated as potential imaging biomarkers of EGFR/KRAS-mutant or TKI-sensitive NSCLC. However, they remain investigational and have not been approved as substitutes for tissue- or plasma-based genotyping [58,59,60,61].

The key question for targeted therapies within precision oncology is no longer whether these actionable mutations exist in NSCLC, but whether identifying and targeting them can meaningfully impact recurrence patterns and affect long-term survival. Equally important is defining the optimal integration and sequencing of these agents within multimodal treatment strategies. Importantly, newer technologies should currently be considered complementary to, rather than a replacement for, tissue- or plasma-based molecular evaluation. Potential future applications include multimodal risk modeling, or selected circumstances in which tissue is limited. However, no validated radiogenomic tool currently independently directs perioperative NSCLC treatment. Prospective, multi-institutional validation and demonstration that model use results in clinically meaningful changes or therapeutic decisions are prerequisites for routine implementation.

## 6. Implementation Gaps: Testing Disparities in Real-World Practice

While promising, the translational pathway from initial discovery to routine clinical implementation remains incomplete. Real-world data reveal substantial variability in biomarker testing rates across institutions and patient populations, with testing rates falling below 55% among uninsured patients and those in lower socioeconomic strata [62,63]. Despite guideline recommendations for universal molecular profiling, only approximately 39 to 46% of patients undergo comprehensive testing for all recommended biomarkers, reflecting operational barriers, communication and knowledge gaps, and barriers with regard to access to care and funding [62,64,65]. These gaps carry critical clinical significance in the perioperative window, when treatment selection may depend on molecular results prior to the initiation of systemic therapy rather than post-recurrence.

An important consideration when evaluating the statistics presented on real-world utilization of molecular profiling is that many of the studies are disproportionately drawn from mixed-stage populations, metastatic NSCLC, and/or community-practice cohorts [62,66]. However, the structural barriers they identify, including inadequate tissue sampling, insurance and socioeconomic disparities, incomplete testing, fragmented care, and prolonged turnaround times, remain directly relevant to perioperative practice.

Although dedicated real-world implementation data focused specifically on resectable NSCLC are limited, these inconsistencies in molecular profiling carry particular consequences for patients with early-stage NSCLC, where the timely identification of actionable driver mutations increasingly informs perioperative treatment strategies. Observational data have demonstrated that patients who undergo routine biomarker testing have significantly improved survival (HR 0.74, 95% CI 0.72–0.76 for death) [62]. This association should not be interpreted solely as the direct effect of biomarker-directed therapy. The ability to undergo molecular testing may also function as a surrogate marker for access to comprehensive, guideline-directed cancer care, including adequate tissue acquisition for pathology and testing, medical oncology involvement, multidisciplinary treatment planning, and timely navigation through complex cancer care. The observational survival association should also not be interpreted causally. Tested and untested populations may differ substantially in performance status, treatment eligibility, socioeconomic resources, tissue availability, follow-up, and access to comprehensive oncology care. Immortal-time bias may be introduced if patients must survive long enough to undergo or complete testing.

Incomplete or delayed molecular testing may also expose patients to inappropriate treatment algorithms. This becomes particularly relevant for patients with EGFR-mutant NSCLC, in whom ICI monotherapy has demonstrated limited efficacy compared with EGFR wild-type disease [67,68]. Treatment sequencing may also introduce toxicity. Dedicated clinical evidence has demonstrated an increased risk of immune-related toxicity when osimertinib is administered shortly after PD-L1 blockade, including pneumonitis, colitis, and hepatitis [69,70,71]. This concern is now reflected in contemporary guidelines and consensus recommendations. The 2026 American Association for Thoracic Surgery Expert Consensus statement for N2+ NSCLC and current National Comprehensive Cancer Network guidelines (NSCL-3, NSCL-5, NSCL-8, NSCL-E-1, NSCL-H) converge on the principle that actionable driver status, particularly EGFR and ALK, should be established before initiating perioperative immunotherapy due to the data suggesting no meaningful efficacy benefit coupled with real risk of harm, both from ICI therapy itself and increased toxicity when the patient subsequently requires mutation-directed TKI therapy [4,70,72].

Failure to identify actionable mutations not only contributes to inequities in clinical trial enrollment and access to precision therapies but also precludes the timely initiation of appropriate adjuvant targeted therapy [67]. For thoracic surgeons, tissue stewardship is therefore one of the most immediate and actionable components of precision oncology. The initial diagnostic strategy should aim to obtain sufficient tissue for histologic classification, PD-L1 assessment, and comprehensive molecular testing. Pathology, pulmonology, interventional radiology, thoracic surgery, and medical oncology should establish comprehensive workflows for perioperative sampling, testing, and evaluation.

The emergence of these therapies and novel strategies makes turnaround time increasingly important in these workflows, as relevant treatment decisions often occur prior to surgery. Delays in testing, incomplete biomarker panels, and disparities driven by demographic or systemic factors risk undermining the full potential of precision oncology. Reflex biomarker testing and sequencing at diagnosis, coordinated multidisciplinary care, and streamlined pathology workflows all represent essential components of modern thoracic oncology practice.

## 7. Minimal Residual Disease (MRD): Molecular Prognostication and Surveillance in Resected NSCLC

While genomic profiling at diagnosis may guide initial therapeutic strategy, emerging technologies such as circulating tumor DNA (ctDNA) are expanding the role of biomarkers beyond treatment selection to surveillance. As an example, ctDNA-based minimal residual disease (MRD) detection is positioned to further redefine the management of early-stage NSCLC. The landmark 2017 TRACERx prospective observational cohort provided critical insights into NSCLC tumor evolution, intratumor heterogeneity, and the emerging role of ctDNA in MRD detection [73]. TRACERx data showed that ctDNA detection can precede radiographic recurrence by a median of 70 days [74,75,76]. In the 2023 TRACERx analysis, landmark plasma samples collected within 120 days after surgery detected ctDNA in 49% of patients who ultimately experienced clinical relapse; serial surveillance identified impending relapse in an additional 20% of patients who had initially been ctDNA-negative. These results demonstrate the potential advantage of longitudinal testing but also illustrate that a single negative postoperative assay cannot exclude future recurrence [76]. The ADAURA MRD analysis published by Herbst and colleagues in 2025 confirmed that ctDNA-based MRD detection precedes imaging-detected DFS events by a median of 4.7 months (95% CI, 2.2–5.6 months) [77].

Postoperative ctDNA positivity also carries substantial prognostic significance. A 2025 meta-analysis by Zhang and colleagues revealed that postoperative ctDNA-positive patients have significantly worse recurrence-free (HR 6.05, 95% CI 4.48–8.18, *p* < 0.01) and overall survival (HR 4.53, 95% CI 2.56–8.02, *p* < 0.01) [78]. These estimates should nevertheless be interpreted in the context of heterogeneity across assay platforms, timing of sampling, stage, adjuvant treatment, definitions of MRD positivity, and study populations. Strong prognostic association does not yet establish MRD as a fully validated predictive biomarker. Ongoing specificity concerns, such as clonal hematopoiesis, variant contamination, and unstandardized positivity thresholds, still limit control of false positives [79].

Current prospective trials are focused on evaluating whether ctDNA-guided therapy escalation or de-escalation can further personalize adjuvant therapy strategies and may ultimately redefine how recurrence risk is assessed and managed [80,81]. Until these studies demonstrate improved clinical outcomes, postoperative ctDNA positivity alone should not mandate escalation of systemic therapy outside a clinical trial, and ctDNA negativity should not be used routinely to omit or de-escalate therapy where otherwise indicated.

## 8. Discussion: Implications of Biomarker Integration for the Thoracic Surgeon

The central clinical challenge in perioperative precision oncology is no longer simply identifying molecular abnormalities but ensuring that clinically relevant results are available before the treatment decision they may change. EGFR and ALK now have phase III evidence supporting genotype-directed adjuvant therapy, while many additional alterations identified by broad sequencing remain therapeutically actionable principally in advanced disease. Likewise, perioperative immune checkpoint blockade has established efficacy in appropriately selected resectable NSCLC, making early molecular characterization essential when driver status may alter whether immunotherapy or targeted therapy is pursued. For the thoracic surgeon, the most immediate implications of precision oncology relate to treatment sequencing, adequate diagnostic tissue sampling, and supporting a comprehensive multidisciplinary workflow.

With regard to operative management, no validated evidence shows that EGFR, ALK, KRAS, postoperative ctDNA status, or another molecular subtype should independently determine the choice between lobectomy and an otherwise oncologically appropriate sublobar resection. Contemporary evidence supporting sublobar resection, including JCOG0802/WJOG4607L and CALGB/Alliance 140503, relies primarily on tumor size, radiographic and anatomic characteristics, nodal assessment, and patient factors rather than molecular subtype [82,83]. The same principles apply to lymph node evaluation, wherein biomarker status does not justify compromising oncologic nodal assessment intraoperatively.

## 9. Future Directions

Several questions remain central to the next phase of perioperative precision oncology. For established targeted therapies, the optimal duration of treatment, consequences of treatment discontinuation, long-term toxicity, cost-effectiveness, and identification of patients deriving the greatest absolute benefit remain incompletely defined. Integrating oncogenic driver status, co-mutation patterns, pathologic response, and ctDNA dynamics may ultimately enable more biologically informed treatment selection, but such approaches require prospective validation before routine implementation. With regard to radiogenomics, future studies should prioritize prospective multi-institutional external validation, standardized image acquisition and feature extraction, and transparent reporting of calibration and discrimination. Most importantly, studies must define what clinical decisions radiogenomic information changes in an era when tissue or plasma genotyping remains required.

As a whole, advances in these facets of perioperative NSCLC management must determine whether these scientifically valid markers produce a meaningful population-level benefit, including evaluation of tissue stewardship, equitable access, and multidisciplinary care. Despite promising preclinical and early clinical data, these advances in clinical oncology cannot meaningfully improve patient outcomes if clinically actionable information is unavailable when treatment decisions are made.

## 10. Conclusions: Working Toward a Fully Integrated Model of Care

The expanding body of literature on biomarker-driven precision oncology in the management of NSCLC reflects a fundamental shift in treatment goals. Management is no longer defined primarily by the resection of visible disease, but increasingly by efforts to anticipate, prevent, and mitigate recurrence through molecularly informed strategies. The challenge for providers and investigators now lies in integration. For patients to truly reap the benefits of these novel technologies, universal molecular testing must advance in parallel alongside rigorous validation of emerging biomarkers and targeted therapies. Precision oncology must be systematically integrated within existing surgical and oncologic care pathways. For thoracic surgeons, the dynamic landscape of perioperative NSCLC management primarily impacts the perioperative workflow. Surgeons increasingly share responsibility for adequate diagnostic testing, multidisciplinary review of relevant results, and appropriate sequencing of surgical therapy.

As these studies collectively illustrate, the question is no longer whether these biomarkers are relevant in the management of NSCLC, but how comprehensively and equitably they may be implemented in clinical practice so these advances may translate into meaningful and durable survival benefits for this patient population.

## Data Availability

No new data were created or analyzed in this study. Data sharing is not applicable to this article.

## Abbreviations

The following abbreviations are used in this manuscript:

NSCLC	Non-small-cell lung cancer
EGFR	Epidermal growth factor receptor
ICI	Immune checkpoint inhibitor
ALK	Anaplastic lymphoma kinase
KRAS	Kirsten Rat Sarcoma
DFS	Disease-free survival
OS	Overall survival
TKI	Tyrosine kinase inhibitor
CNS	Central nervous system
CT	Computed tomography
HR	Hazard ratio
ctDNA	Circulating tumor DNA
MRD	Minimal residual disease
